# Trunk muscle activation patterns during active hip abduction test during remission from recurrent low back pain: an observational study

**DOI:** 10.1186/s12891-021-04538-5

**Published:** 2021-08-09

**Authors:** Tadanobu Suehiro, Hiroshi Ishida, Kenichi Kobara, Hiroshi Osaka, Chiharu Kurozumi

**Affiliations:** 1 Department of Physical Therapy, Kawasaki University of Medical Welfare, 288, 701-0193 Matsushima, Kurashiki City, Japan; 2 Department of Occupational Therapy, Kawasaki University of Medical Welfare, 288, 701-0193 Matsushima, Kurashiki City, Japan

**Keywords:** Active hip abduction test, Low back pain, Electromyography, Trunk muscle activation pattern, Transversus abdominis

## Abstract

**Background:**

The active hip abduction test (AHAbd) is widely used to evaluate lumbopelvic stability, but the onset of trunk muscle activation during the test in individuals with recurrent low back pain (rLBP) has not been investigated so far. It is important to investigate the pattern of trunk muscle activation during the AHAbd test to provide insight into the interpretation of observation-based assessment results; this may help to create exercise therapy interventions, from a movement control perspective, for people seeking treatment for rLBP. The purpose of this study was to compare the timing of trunk muscle activation between individuals with and without rLBP and to assess potential differences.

**Methods:**

Seventeen subjects in remission from rLBP and 17 subjects without rLBP were recruited. We performed surface electromyography of the transversus abdominis/internal abdominal oblique, external oblique, erector spinae, and gluteus medius muscles during the AHAbd test on both sides. The onset of trunk muscle activation was calculated relative to the prime mover gluteus medius. The independent-samples t- and Mann-Whitney U tests were used to compare the onset of trunk muscle activation between the two groups.

**Results:**

The onset of transversus abdominis/internal abdominal oblique activation on the ipsilateral (right AHAbd: −3.0 ± 16.2 vs. 36.3 ± 20.0 msec, left AHAbd: −7.2 ± 18.6 vs. 29.6 ± 44.3 ms) and contralateral sides (right AHAbd: −11.5 ± 13.9 vs. 24.4 ± 32.3 ms, left AHAbd: −10.1 ± 12.5 vs. 23.3 ± 17.2 ms) and erector spinae on the contralateral side (right AHAbd: 76.1 ± 84.9 vs. 183.9 ± 114.6 ms, left AHAbd: 60.7 ± 70.5 vs. 133.9 ± 98.6 ms) occurred significantly later in individuals with rLBP than in individuals without rLBP (p < 0.01). During the left AHAbd test, the ipsilateral erector spinae was also activated significantly later in individuals with rLBP than in individuals without rLBP (71.1 ± 80.1 vs. 163.8 ± 120.1 ms, p < 0.05). No significant difference was observed in the onset of the external oblique activation on the right and left AHAbd tests (p > 0.05).

**Conclusions:**

Our results suggest that individuals with rLBP possess a trunk muscle activation pattern that is different from that of individuals without rLBP. These findings provide an insight into the underlying muscle activation patterns during the AHAbd test for people with rLBP and may support aggressive early intervention for neuromuscular control.

## Background

Low back pain (LBP) is a significant musculoskeletal problem worldwide with a lifetime prevalence of up to 65 % [1]. The social and economic impacts of LBP, including the indirect and direct costs, are considerable. While 90 % of LBP episodes resolve spontaneously within 1 month, between 42 and 75 % of people experience a recurrence within 12 months. Recurrent LBP (rLBP) contributes to a higher proportion of work disability and medical and indemnity costs than the initial LBP episode [2].

The stability of the lumbopelvic region is reportedly maintained by passive subsystems such as the vertebrae, facet, and spinal ligament, active subsystems such as the muscles and tendons, and neural control subsystem [3]. Damage to the passive tissues, impairments in neural control mechanisms including neural drive, and impairments in muscle and tendons, such as muscle weakness, decrease the stability of the lumbopelvic region [3].

Patients with LBP have been reported to display changed neuromuscular activity such as delayed local muscle activation and greater co-contraction of the abdominal and back muscles [4–7]. Some studies suggest that these changes extend beyond the duration of a painful episode and could lead to long-term consequences [8, 9], as the pain may recur due to the increased loading of the spine [10, 11].

The active hip abduction (AHAbd) test evaluates a patient’s movement control in the lumbopelvic region during active abduction of the hip while the patient lies on their side with both legs extended. It assesses the patient’s ability to maintain lumbopelvic alignment against the rotational torque of the pelvis and trunk caused by gravity [12]. This test has been reported to identify people who are at risk for developing LBP during prolonged standing [13]. People who developed LBP during prolonged standing have shown decreased movement control of the lumbopelvic region during the AHAbd test compared to people who did not develop LBP, manifesting as lumbopelvic rotation and asymmetric lumbopelvic movement [13, 14]. A previous study compared the onset of trunk muscle activation during the AHAbd test in patients with LBP to that in individuals without LBP and reported a delayed onset of the ipsilateral internal oblique, ipsilateral external oblique (EO), and contralateral erector spinae (ES) muscles in patients with LBP [15]. However, this study did not specify the speed of lower extremity movement during the AHAbd test. The speed of limb movement has been reported to affect the frequency and variability of the electromyography (EMG) response of the deep trunk muscles [16, 17]. Therefore, the significance of changes in muscle activation onset in individuals with LBP during the AHAbd test remains controversial. In addition, EMG onset of the deep trunk muscles has been reported to be delayed in individuals in remission from rLBP during limb movement [18]. The muscle activation onset during the AHAbd test in individuals with rLBP has not been investigated so far, even though changes in muscle activation patterns in LBP are dependent on the task [19]. Furthermore, delayed activation of the deep muscles of the trunk has been reported to be associated with excessive lumbopelvic movement during prone hip extension in healthy individuals [20]. Excessive lumbopelvic movement can lead to a high concentration of stress in the lumbar vertebrae and surrounding soft tissues [21, 22]. Investigating the timing of the trunk muscle activation during the AHAbd test is important to provide insight into the interpretation of the observation-based assessment results.

The purpose of this study was to compare the onset of trunk muscle activation during the AHAbd test between individuals with and without rLBP and to clarify any changes in the timing of trunk muscle activation in individuals with rLBP. We hypothesised that individuals with rLBP would demonstrate different muscle activation patterns compared to individuals without rLBP during the AHAbd test.

## Methods

### Design

This study has a cross-sectional design.

### Participants

Thirty-four volunteers (17 with rLBP and 17 without rLBP) between the ages of 20 and 40 were recruited through poster advertisements at our university. Subjects with rLBP had to have had two or more episodes of LBP over the last year, localised between the level of the 12th thoracic vertebra and the horizontal gluteal fold that restricted their leisure, work, or sports activities [23, 24]. Subjects were excluded if they had neurological disorders, a history of fractures or surgery in the hip joints or spine, or a passive range of hip abduction motion of less than 30°. Each participant underwent an interview-based screening for the inclusion and exclusion criteria. The passive range of motion of hip abduction was examined using a goniometer with the participant in a supine position with their knee extended prior to data collection to confirm exclusion criteria. At the time of testing, all subjects were pain-free.

We explained the purpose, procedures, and any potential benefits and risks of the study to each subject. All participants read the protocol and provided written informed consent. Ethical approval was obtained from the Ethics Committee of the Kawasaki University of Medical Welfare (study number: 19 − 010, 20 − 002).

### LBP and LBP-related disability assessment

Before data collection, subjects with rLBP were asked to score the current severity of their pain, along with that experienced over the previous week, using a numeric rating scale from 0 (“no pain”) to 10 (“worst possible pain”). In addition, the LBP-related disability was assessed using the Japanese version of the Oswestry Disability Index, which ranges from 0 (no disability) to 100 (maximum disability) [25].

### Electromyography

Muscle activity during the AHAbd test was measured using a wireless surface EMG (MQ-Air; Kissei Comtec Co., Ltd., Nagano, Japan) with a 1,000-Hz sampling frequency and a surface-type electrode (Ambu® BlueSensor R; Ambu A/S, Ballerup, Denmark). After the skin had been shaved and cleaned with alcohol, the electrodes were applied with a 2.5-cm inter-electrode distance parallel to the muscle fibres of the following muscles: the EO (15 cm lateral to the umbilicus), transverse abdominis/internal oblique (TrA/IO, 2 cm inferior and medial to the anterior superior iliac spine), and ES (3 cm lateral to spinous process of L1) bilaterally, and the gluteus medius (midpoint of a line from the crista iliaca to the greater trochanter) on the side of the hip abduction. A reference electrode was attached above the second sacral vertebra.

### AHAbd test procedure

**Fig. 1:**
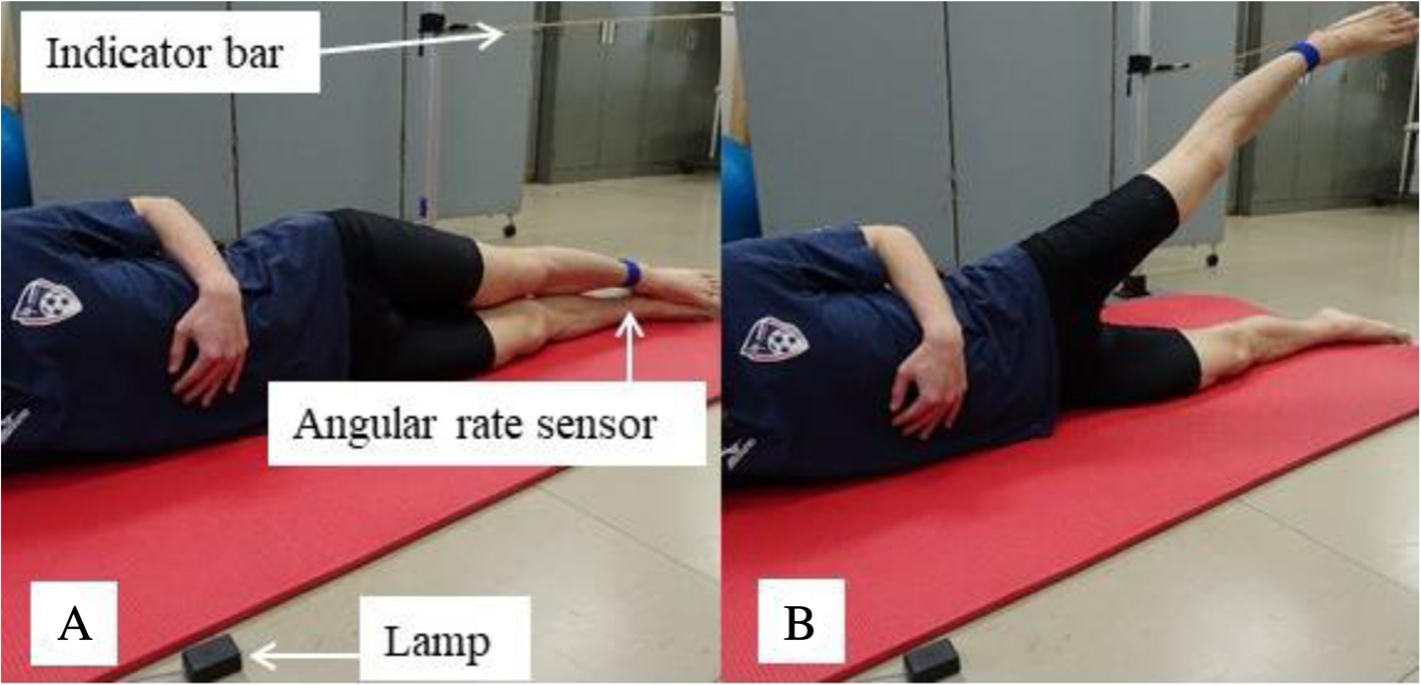
Active hip abductiontest used in this study.When the lamp was turned on, participants had to raise the legas fast as possible until it touched the indicator bar while minimising movementof pelvis and trunk and keeping their knee straight and the leg in line withthe trunk. **A**: Starting position. **B**: Ending position

The subjects were positioned lying on their sides with both legs extended and aligned with the shoulder, trunk, and pelvis in the front plane (Fig. 1). They placed their free arm on their chest to ensure that they did not use it for balance during the AHAbd test. An indicator bar was set such that the participants did not raise their leg further than 30° of hip abduction. A lamp was placed in front of their eyes. When it was turned on, subjects had to raise their upper leg in line with their trunk and with a straight knee as fast as possible until it touched the indicator bar, while minimising any movement of pelvis and trunk. Subjects practiced the AHAbd test a few times to familiarise themselves with the movement.

### Leg movement speed during AHAbd

The speed (°/sec) of the leg movement during AHAbd was measured using an angular rate sensor (MVP-RF10-AC; MicroStone Corporation, Nagano, Japan), which was attached to the ankle of the abducted leg. All subjects performed three AHAbd tests with each leg in random order.

### Data processing

EMG signals were band-pass filtered (10–500 Hz). We then applied the Teager-Kaiser energy operator that was reported to reduce the mean detection error for the determination of the onset of muscle activity [26].

The discrete Teager-Kaiser energy operator Ψ was expressed as:

$$ \psi \left[x(t)\right]={x}^2(t)-x\left(t+1\right)\ast \left(t-1\right), $$

where [x(t)] is the EMG value at the point in time t. Following that, the full-wave rectification was performed.

Visual detection of muscle activation has previously been shown to be reliable and was preferred to the computer-based method, as it is less affected by the rate of increasing muscle activation and increased background activity [27]. Therefore, the onset of muscle activation was determined by visual analysis as the first increase in EMG activity above the baseline [26, 28, 29]. An investigator who was blinded to the subject’s group evaluated the onset of trunk muscle activation. The onset of trunk muscle activation was expressed relative to the prime mover (gluteus medius muscle) using the following equation:

Relative onset of trunk muscle activation = onset of trunk muscle activation – gluteus medius activation.

Thus, a positive value meant that the trunk muscles were activated after the gluteus medius.

### Statistical analysis

We estimated the sample size based on a pilot study in five subjects with rLBP and five subjects without rLBP. G-power 3.1 software [30] was used to calculate the required sample size based on a power of 0.9, an effect size of 0.91, and a significance level of 0.05. This resulted in the need for 17 subjects in each group.

Statistical analyses were performed using IBM SPSS Statistics for Windows, version 23 (IBM Corp., Armonk, NY, USA). Variables were presented as the mean ± standard deviation. The Shapiro-Wilk test of normality was used for all continuous variables. The independent-samples t-test was used to investigate the potential differences between the rLBP and non-LBP groups in terms of the participant demographics, relative onset of trunk muscle activation, and leg movement speed during the AHAbd test. In the case of not normally distributed data, the Mann-Whitney U test was employed. The level of statistical significance was set at p < 0.05.

## Results

### Demographics

The demographics of the rLBP and non-LBP groups are shown in Table 1. There were no significant differences in age or body mass index between the two groups.

**Table 1:** Demographics of subjects with and without recurrent lower back pain

	Non-LBP group	rLBP group	p-value
Subjects (n)	17	17	
Sex (male:female)	14:3	14:3	
Age (years)	21.6 ± 3.5	22.1 ± 5.0	0.69
Body mass index	21.3 ± 3.0	21.5 ± 2.4	0.79
Oswestry Disability Index (%)		10.1 ± 6.5	
Previous week’s average pain score		3.4 ± 1.5	
Current pain score		0 ± 0	
Time since initial LBP episode (years)		5.4 ± 4.7	

Values are presented as the mean ± standard deviation. rLBP: recurrent low back pain

### Speed of leg movement during AHAbd test

There were no significant differences in the speed of the leg movements between the non-LBP and rLBP groups during the right (non-LBP group: 25.5°±7.4°/sec, rLBP group: 29.9°±6.9°/sec; power = 0.42, effect size d = 0.62, p = 0.08) and left (non-LBP group: 24.6°±5.9°/sec, rLBP group: 28.2°±7.7 °/sec; power = 0.31, effect size d = 0.53, p = 0.21) AHAbd tests.

### Relative onset of trunk muscle activation during AHAbd test

The contralateral side of the trunk muscle was defined as the contralateral side of the leg moving side, and the ipsilateral side of the trunk muscle was defined as the ipsilateral side of the leg moving side.

**Fig. 2:**
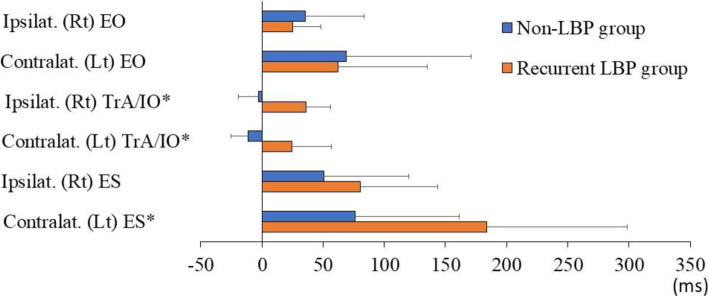
The relative onset oftrunk muscle activation during the right active hip abduction test.Asterisks indicate significantdifferences between groups. Positive values mean that the respective trunkmuscle was activated after the gluteus medius. Ipsilat, ipsilateral; Contralat,contralateral; EO, external oblique; TrA/IO, transverse abdominis/internaloblique; ES, erector spinae; Rt, right; Lt, left; LBP, low back pain

**Fig. 3:**
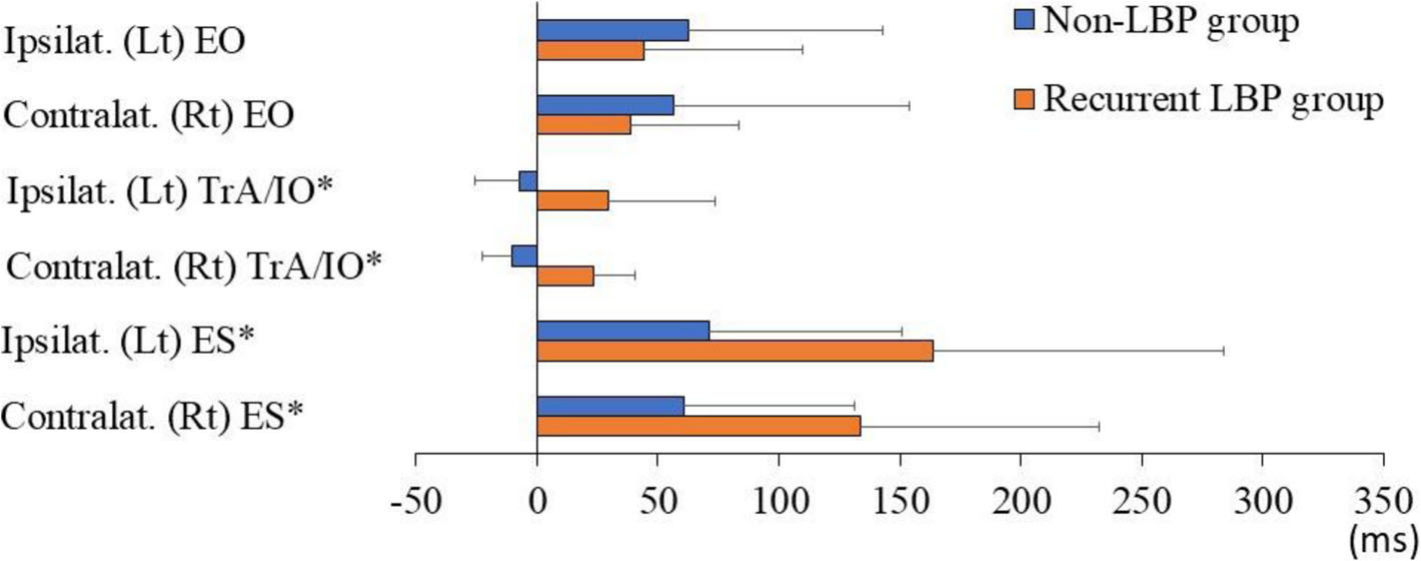
The onset of trunkmuscle activation during the left active hip abduction test. Asterisks indicatesignificant differences between groups. Positive values mean that the trunkmuscle was activated after the gluteus medius. Ipsilat, ipsilateral; Contralat,contralateral; EO, external oblique; TrA/IO, transverse abdominis/internaloblique; ES, erector spinae; Rt, right; Lt, left; LBP, low back pain

The relative onset of trunk muscle activation was compared between the two groups during the right and left AHAbd tests (Table 2; Figs. 2 and 3). During the right AHAbd test, the onset of the ipsilateral (non-LBP group: −3.0 ± 16.2 ms, rLBP group: 36.3 ± 20.0 ms; power = 1.00, effect size d = 2.16, p < 0.001) and contralateral (non-LBP group: −11.5 ± 13.9 ms, rLBP group: 24.4 ± 32.3 ms; power = 0.98, effect size d = 1.45, p < 0.001) TrA/IO and the contralateral ES (non-LBP group: 76.1 ± 84.9 ms, rLBP group: 183.9 ± 114.6 ms; power = 0.84, effect size d = 1.07, p = 0.001) occurred significantly later in the rLBP group than in the non-LBP group. No significant differences in the onset of the ipsilateral and contralateral EO and ipsilateral ES were found between the two groups.

**Table 2:** Onset of muscle activation during the active hip abduction test

	Non-LBP group (n = 17)	rLBP group (n = 17)	p-value	Effect size (d)	Power
Right					
Ipsilat external oblique	35.5 ± 47.9	24.8 ± 23.6	0.877	0.28	0.28
Contralat external oblique	68.6 ± 102.3	61.9 ± 73.4	0.945	0.08	0.05
Ipsilat TrA/IO^*^	-3.0 ± 16.2	36.3 ± 20.0	< 0.001	2.16	1.00
Contralat TrA/IO^*^	-11.5 ± 13.9	24.4 ± 32.3	< 0.001	1.45	0.98
Ipsilat erector spinae	50.8 ± 69.4	80.2 ± 63.6	0.139	0.44	0.23
Contralat erector spinae^*^	76.1 ± 84.9	183.9 ± 114.6	0.001	1.07	0.84
Left					
Ipsilat external oblique	62.8 ± 80.3	44.2 ± 65.4	0.617	0.25	0.25
Contralat external oblique	56.3 ± 97.9	38.9 ± 44.8	0.877	0.23	0.10
Ipsilat TrA/IO^*^	-7.2 ± 18.6	29.6 ± 44.3	< 0.001	1.08	0.87
Contralat TrA/IO^*^	-10.1 ± 12.5	23.3 ± 17.2	< 0.001	2.22	1.00
Ipsilat erector spinae^*^	71.1 ± 80.1	163.8 ± 120.1	0.011	0.91	0.71
Contralat erector spinae^*^	60.7 ± 70.5	133.9 ± 98.6	0.008	0.85	0.65

Values are mean ± standard deviation. *P-value < 0.05. rLBP: recurrent low back pain; Ipsilat: ipsilateral; Contralat: contralateral; TrA/IO: transversus abdominis/internal abdominal oblique

During the left AHAbd test, the onset of the ipsilateral (non-LBP group: −7.2±18.6 ms, rLBP group: 29.6±44.3 ms; power=0.87, effect size d=1.08, p<0.001) and contralateral (non-LBP group: −10.1±12.5 ms, rLBP group: 23.3±17.2 ms; power=1.00, effect size d=2.22, p<0.001) TrA/IO and ipsilateral (non-LBP group: 71.1±80.1 ms, rLBP group: 163.8±120.1 ms; power=0.71, effect size d=0.91, p=0.011) and contralateral (non-LBP group: 60.7±70.5 ms, rLBP group: 133.9±98.6 ms; power=0.65, effect size d=0.85, p=0.008) ES occurred significantly later in the rLBP group than in the non-LBP group. No significant difference in the onset of the ipsilateral and contralateral EO was found between the two groups.

## Discussion

In this first study investigating potential differences in the onset of trunk muscle activation during the AHAbd test between individuals with and without rLBP, we found that the bilateral TrA/IO and the contralateral ES onset in individuals with rLBP during the asymptomatic interval occurred later than that in subjects without rLBP. The onset of the ipsilateral ES activation during the left AHAbd test occurred later in individuals with rLBP than in those without rLBP. These findings support our hypothesis that individuals with rLBP demonstrate different trunk muscle activation patterns compared to subjects without rLBP.

Although no significant differences in the speed of the leg movements were found between the two groups, the statistical power was low at 0.42. This had the potential to produce a type 2 error. In this regard, previous research has found that the onset of trunk muscle activation occurred earlier for fast limb movements than for slow limb movements [16]. In this study, even though the speed of leg movement was, on average, 4.4°/sec faster in subjects with rLBP than in those without rLBP, the onset of bilateral TrA/IO and contralateral ES occurred significantly later in the subjects with rLBP than in those without rLBP. Therefore, it is unlikely that the onset of trunk muscle activation in both groups was affected by the speed of the movement. This result indicates that subjects with rLBP used different trunk muscle activation patterns than those with rLBP, even though the subjects with rLBP were pain-free during the test and had minimal disabilities with ODI scores of 10 %. Our results are consistent with those reported in previous studies comparing individuals with rLBP who had delayed TrA/IO onset with healthy subjects during limb movement and lifting tasks [9, 31].

The AHAbd test is also used to assess the maintenance of neutral lumbopelvic alignment against the rotational torque of the pelvis and trunk. TrA/IO activation has been reported to contribute to the control of intervertebral movements in the transverse plane and counterbalance the rotational torque during limb movement [32–34]. Osuka et al. [35] found that the onset of TrA/IO activation occurred significantly later in individuals with LBP than in individuals without LBP during tasks with trunk rotation torque, but not during the task without torque. They suggested that the delayed TrA/IO activation during a task with trunk rotation torque is a characteristic of chronic LBP. In addition, poor proprioception, injury, fear of pain, and reorganisation of the TrA representation in the motor cortex may alter motor control [31, 36–40]. Therefore, although proprioception, fear of pain and motor cortex reorganisation were not measured in this study, the late TrA/IO activation during the AHAbd test in individuals with rLBP may be related to poor proprioception, fear of pain, reorganisation of the motor cortex, and the challenge of maintaining the pelvis and trunk in a neutral position against the rotational torque. TrA/IO contraction reportedly contributes to controlling the intersegmental lumbar spine motion [21]. Thus, the delayed TrA/IO activation may lead to a reduce capability in controlling intersegmental motion at the initiation of the leg movement during the AHAbd test; this may contribute to repetitive microtrauma to the spinal tissue, leading to recurring episodes of LBP [41, 42].

In this study, the onset of contralateral ES activation during the right AHAbd test and the onset of bilateral ES activation during the left AHAbd test was significantly delayed in individuals with rLBP compared to that in individuals without rLBP. Previous studies found that individuals who developed LBP symptoms while standing displayed increased lumbopelvic movement and less symmetry in the timing of lateral pelvic tilt compared to healthy subjects who did not develop LBP symptoms during the AHAbd test [13, 14]. The global muscles such as the ES control spinal orientation, balance the external loads applied to the spine, and provide general trunk stability [21]. In addition, the activity of the contralateral ES contributes to the prevention of excessive lateral pelvic tilt during the AHAbd test. Therefore, the delayed ES activation in the AHAbd test in this study could have led to excessive movement of the lumbopelvic region; however, the motion of lumbopelvic region was not measured in this study. These changes may increase the likelihood of both the recurrence and chronicity of LBP because excessive movement of the lumbopelvic region can result in a concentration of spinal tissue stress such as abnormal deformation of pain sensitive structures and ligaments or stretching and compression of neural structures [21].

The method for conducting the AHAbd test used in this study differed in some aspects from previously reported methods. In previous AHAbd tests, the subject performed active hip abduction at a natural speed with up to 80 % of their available range of motion [12, 13, 15, 43]. However, in this study, the subject performed active hip abduction of 30°, as fast as possible, because the speed of limb movement has been reported to affect the onset of trunk muscle activation. Therefore, some subjects in this study needed to abduct the hip to near the end of their range of motion. Due to these differences in method, the AHAbd test used in this study was more difficult to maintain in terms of the frontal plane alignment of the pelvis, due to larger internal perturbations than the methods used in the previous study [12, 13, 15, 43]. Therefore, subjects were required to practice to become familiar with the movements before data collection; no such practice exercises were performed by the subjects in the previous study. The differences in the AHAbd test method limit comparisons with previous studies [12, 13, 15, 43], and because the practice before data collection provided subjects with the opportunity to learn the exercises, the scores of the test could not be evaluated in this study.

We observed altered muscle activation strategies in individuals with rLBP attempting to maintain lumbopelvic control during the AHAbd test. Generally, clinicians visually assess the aberrant movements of the lumbopelvic region during the AHAbd test, but do not necessarily have insight into the underlying muscle activation pattern in individuals in remission for rLBP during the AHAbd test. The current findings may be useful in devising exercise therapy interventions for people seeking treatment for rLBP from a movement control perspective.

This study has some limitations. First, the sample size was small, and the statistical power was low for variables that were not statistically significant, such as the onset of bilateral EO activation during right and left AHAbd, or onset of ipsilateral ES activation during right AHAbd. Therefore, even though the value of the effect size for these variables was low (d = 0.08–0.44), there was a potential for type II errors. Second, we used surface EMG, which cannot assess the TrA/IO separately, while the activation of other muscles might interfere with the measurements. However, previous studies have established the differences in the onset of TrA/IO activation between individuals with and without LBP using surface EMG and also recorded TrA/IO activation independently of other muscles [9, 44, 45]. Thus, we believe that the difference in the onset of TrA/IO activation between our two study groups is valid. Third, the quadratus lumborum is known to contribute to lumbopelvic stability during the AHAbd test but it cannot be measured using surface EMG because of its deep location. Finally, the subjects with rLBP in this study were younger individuals with minimal disabilities, and the observed task was the AHAbd test in the open kinetic chain movement. Therefore, the findings of this study cannot be generalized to middle-aged and older individuals, those with severe disabilities, and to functional tasks in the closed kinetic chain movement. However, the AHAbd test is generally strong for predicting LBP during standing which is a functional task [13], although it is not a functional test itself. Future studies are necessary to investigate whether treatment aimed at improving muscle activation patterns can help prevent recurrence in individuals with LBP.

## Conclusions

The onset of bilateral TrA/IO activation and contralateral ES occurred later in individuals with rLBP compared with that in individuals without rLBP during the right and left AHAbd tests, and the onset of ipsilateral ES activation during the left AHAbd test occurred later in individuals with rLBP compared to that in individuals without rLBP. Our results show that individuals with rLBP have a muscle activation pattern that differs from that of individuals without rLBP. These findings provide an insight into the underlying muscle activation pattern during the AHAbd test for people with rLBP. In addition, given the high recurrent rate of LBP, these findings may support aggressive early intervention for neuromuscular control.  

## Data Availability

The datasets used and/or analysed during the current study are available from the corresponding author upon reasonable request.

## Abbreviations

AHAbd: Active hip abduction
EMG: Electromyography
EO: External oblique
ES: Erector spinae
LBP: Low back pain
rLBP: Recurrent low back pain
TrA/IO: Transverse abdominis/internal oblique

## References

[1] Manchikanti L (2000). Epidemiology of low back pain. Pain Physician.

[2] Wasiak R, Kim J, Pransky G (2006). Work disability and costs caused by recurrence of low back pain: longer and more costly than in first episodes. Spine.

[3] Panjabi MM (1992). The stabilizing system of the spine. Part I. Function, dysfunction, adaptation, and enhancement. J Spinal Disord.

[4] Marshall P, Murphy B (2010). Delayed abdominal muscle onsets and self-report measures of pain and disability in chronic low back pain. J Electromyogr Kinesiol.

[5] Suehiro T, Mizutani M, Ishida H, Kobara K, Osaka H, Watanabe S (2015). Individuals with chronic low back pain demonstrate delayed onset of the back muscle activity during prone hip extension. J Electromyogr Kinesiol.

[6] Radebold A, Cholewicki J, Panjabi MM, Patel TC (2000). Muscle response pattern to sudden trunk loading in healthy individuals and in patients with chronic low back pain. Spine.

[7] van Dieën JH, Cholewicki J, Radebold A (2003). Trunk muscle recruitment patterns in patients with low back pain enhance the stability of the lumbar spine. Spine.

[8] D’Hooge R, Hodges P, Tsao H, Hall L, Macdonald D, Danneels L (2013). Altered trunk muscle coordination during rapid trunk flexion in people in remission of recurrent low back pain. J Electromyogr Kinesiol.

[9] Suehiro T, Ishida H, Kobara K, Osaka H, Watanabe S (2018). Altered trunk muscle recruitment patterns during lifting in individuals in remission from recurrent low back pain. J Electromyogr Kinesiol.

[10] Hodges PW, Tucker K (2011). Moving differently in pain: A new theory to explain the adaptation to pain. Pain.

[11] van Dieën JH, Flor H, Hodges PW. Low-back pain patients learn to adapt motor behavior with adverse secondary consequences. Exerc Sport Sci Rev. 2017;45:223–9. 10.1249/JES.0000000000000121 28704216

[12] Davis AM, Bridge P, Miller J, Nelson-Wong E (2011). Interrater and intrarater reliability of the active hip abduction test. J Orthop Sports Phys Ther.

[13] Nelson-Wong E, Flynn T, Callaghan JP (2009). Development of active hip abduction as a screening test for identifying occupational low back pain. J Orthop Sports Phys Ther.

[14] Sorensen CJ, Johnson MB, Norton BJ, Callaghan JP, Van Dillen LR (2016). Asymmetry of lumbopelvic movement patterns during active hip abduction is a risk factor for low back pain development during standing. Hum Mov Sci..

[15] Nelson-Wong E, Poupore K, Ingvalson S, Dehmer K, Piatte A, Alexander S (2013). Neuromuscular strategies for lumbopelvic control during frontal and sagittal plane movement challenges differ between people with and without low back pain. J Electromyogr Kinesiol.

[16] Hodges PW, Richardson CA (1997). Relationship between limb movement speed and associated contraction of the trunk muscles. Ergonomics.

[17] Hodges PW, Richardson CA (1999). Altered trunk muscle recruitment in people with low back pain with upper limb movement at different speeds. Arch Phys Med Rehabil.

[18] MacDonald D, Moseley LG, Hodges PW (2009). Why do some patients keep hurting their back? Evidence of ongoing back muscle dysfunction during remission from recurrent back pain. Pain.

[19] Hodges PW, Cholewicki J, Van Dieën JH (2013). Spinal control: the rehabilitation of back pain: state of the art and science.

[20] Tateuchi H, Taniguchi M, Mori N, Ichihashi N (2012). Balance of hip and trunk muscle activity is associated with increased anterior pelvic tilt during prone hip extension. J Electromyogr Kinesiol.

[21] Richardson C, Hodges P, Hides J (2004). Therapeutic exercise for lumbopelvic stabilization.

[22] Gardner-Morse M, Stokes IA, Laible JP (1995). Role of muscles in lumbar spine stability in maximum extension efforts. J Orthop Res.

[23] Macdonald DA, Dawson AP, Hodges PW (2011). Behavior of the lumbar multifidus during lower extremity movements in people with recurrent low back pain during symptom remission. J Orthop Sports Phys Ther.

[24] Ampomah K, Amano S, Wages NP, Volz L, Clift R, Ludin AFM (2019). Blood flow-restricted exercise does not induce a cross-transfer of effect: A randomized controlled trial. Med Sci Sports Exerc.

[25] Fairbank JC, Pynsent PB (2000). The Oswestry disability index. Spine.

[26] Solnik S, Rider P, Steinweg K, DeVita P, Hortobágyi T (2010). Teager-Kaiser energy operator signal conditioning improves EMG onset detection. Eur J Appl Physiol.

[27] Hodges PW, Bui BH (1996). A comparison of computer-based methods for the determination of onset of muscle contraction using electromyography. Electroencephalogr Clin Neurophysiol.

[28] Horak FB, Esselman P, Anderson ME, Lynch MK (1984). The effects of movement velocity, mass displaced, and task certainty on associated postural adjustments made by normal and hemiplegic individuals. J Neurol Neurosurg Psychiatry.

[29] Brown JE, Frank JS (1987). Influence of event anticipation on postural actions accompanying voluntary movement. Exp Brain Res.

[30] Faul F, Erdfelder E, Lang AG, Buchner A (2007). G*Power 3: a flexible statistical power analysis program for the social, behavioral, and biomedical sciences. Behav Res Methods.

[31] Tsao H, Galea MP, Hodges PW (2008). Reorganization of the motor cortex is associated with postural control deficits in recurrent low back pain. Brain.

[32] Massé-Alarie H, Beaulieu LD, Preuss R, Schneider C (2015). Task-specificity of bilateral anticipatory activation of the deep abdominal muscles in healthy and chronic low back pain populations. Gait Posture.

[33] Morris SL, Lay B, Allison GT (2012). Corset hypothesis rebutted–transversus abdominis does not co-contract in unison prior to rapid arm movements. Clin Biomech.

[34] Allison GT, Morris SL, Lay B (2008). Feedforward responses of transversus abdominis are directionally specific and act asymmetrically: implications for core stability theories. J Orthop Sports Phys Ther.

[35] Osuka S, Koshino Y, Yamanaka M, Miura T, Saito Y, Ueno R (2019). The onset of deep abdominal muscles activity during tasks with different trunk rotational torques in subjects with non-specific chronic low back pain. J Orthop Sci.

[36] Goossens N, Janssens L, Brumagne S (2019). Changes in the organization of the secondary somatosensory cortex while processing lumbar proprioception and the relationship with sensorimotor control in low back pain. Clin J Pain.

[37] Moseley GL, Nicholas MK, Hodges PW (2004). Does anticipation of back pain predispose to back trouble?. Brain.

[38] Hodges PW, Moseley GL, Gabrielsson A, Gandevia SC (2003). Experimental muscle pain changes feedforward postural responses of the trunk muscles. Exp Brain Res.

[39] Panjabi MM (2006). A hypothesis of chronic back pain: ligament subfailure injuries lead to muscle control dysfunction. Eur Spine J.

[40] Alsubaie AM, Martinez-Valdes E, De Nunzio AM, Falla D (2021). Trunk control during repetitive sagittal movements following a real-time tracking task in people with chronic low back pain. J Electromyogr Kinesiol.

[41] Panjabi MM (1992). The stabilizing system of the spine. Part II. Neutral zone and instability hypothesis. J Spinal Disord.

[42] Hodges PW, Moseley GL (2003). Pain and motor control of the lumbopelvic region: effect and possible mechanisms. J Electromyogr Kinesiol.

[43] Nelson-Wong E, Gallant P, Alexander S, Dehmer K, Ingvalson S, McClenahan B (2016). Multiplanar lumbopelvic control in patients with low back pain: is multiplanar assessment better than single plane assessment in discriminating between patients and healthy controls?. J Man Manip Ther..

[44] Marshall P, Murphy B (2003). The validity and reliability of surface EMG to assess the neuromuscular response of the abdominal muscles to rapid limb movement. J Electromyogr Kinesiol.

[45] Silfies SP, Mehta R, Smith SS, Karduna AR (2009). Differences in feedforward trunk muscle activity in subgroups of patients with mechanical low back pain. Arch Phys Med Rehabil.

